# Population Pharmacokinetic Analysis of Delamanid in Patients with Pulmonary Multidrug-Resistant Tuberculosis

**DOI:** 10.1128/AAC.01202-20

**Published:** 2020-12-16

**Authors:** Xiaofeng Wang, Suresh Mallikaarjun, Ekaterina Gibiansky

**Affiliations:** a Otsuka Pharmaceutical Development and Commercialization, Inc., Rockville, Maryland, USA; b QuantPharm LLC, North Potomac, Maryland, USA

**Keywords:** *Mycobacterium tuberculosis*, delamanid, population pharmacokinetics

## Abstract

A population pharmacokinetic (PopPK) model of delamanid in patients with pulmonary multidrug-resistant tuberculosis (MDR-TB) was developed using data from four delamanid clinical trials. The final PopPK data set contained 20,483 plasma samples from 744 patients with MDR-TB receiving an optimized background regimen (OBR). Delamanid PK was adequately described for all observed dosing regimens and subpopulations by a two-compartment model with first-order elimination and absorption, an absorption lag time, and decreased relative bioavailability with increasing dose. Relative bioavailabilities of 200-mg and higher doses (250 and 300 mg) were 76% and 58% of a 100-mg dose, respectively. Relative bioavailability was 26% higher after evening doses than morning doses and 9% higher in outpatient settings than inpatient settings. The rate of absorption was higher, and lag time was shorter, following a morning dose than an evening dose. Relative bioavailabilities in patients in Northeast Asian and Southeast Asian regions were 53% and 40% higher, respectively, than in patients in non-Asian regions. Apparent clearance was higher (to the power of −0.892) in patients with hypoalbuminemia (albumin levels of <3.4 g/dl). Coadministration of efavirenz in patients with HIV increased delamanid clearance by 35%. Delamanid exposure was not affected by age (18 to 64 years), mild or moderate renal impairment, anti-TB antibiotic resistance status, HIV status, or markers of hepatic dysfunction or by concomitant administration of OBR, lamivudine, tenofovir, pyridoxine, CYP3A4 inhibitors and inducers, or antacids. Model evaluation suggested reasonable model fit and predictive power, indicating that the model should prove reliable to derive PK metrics for subsequent PK/PD analyses.

## Abstract (precis)

A population pharmacokinetic (PopPK) model of delamanid in patients with pulmonary multidrug-resistant tuberculosis (MDR-TB) was developed using data from four delamanid clinical trials. The final PopPK data set contained 20,483 plasma samples from 744 patients with MDR-TB receiving an optimized background regimen (OBR). Delamanid PK was adequately described for all observed dosing regimens and subpopulations by a two-compartment model with first-order elimination and absorption, an absorption lag time, and decreased relative bioavailability with increasing dose.

## TEXT

Tuberculosis (TB) caused an estimated 1.2 million deaths in 2018 among the 10 million people who developed the disease, making it responsible for more deaths globally per year than any other infectious disease (1). Delamanid (Deltyba; Otsuka Pharmaceutical Co., Ltd., Tokushima, Japan) is a bicyclic nitroimidazooxazole compound that inhibits the synthesis of mycolic acids (2), key components of the lipid-rich cell wall of M. tuberculosis (3). Preclinical and clinical studies have demonstrated the efficacy of delamanid against multidrug-resistant tuberculosis (MDR-TB), defined as tuberculosis that is resistant to isoniazid and rifampin (4–9). Currently, delamanid is approved in several countries for adults and in one country (India) for children, as part of an appropriate combination regimen for MDR-TB when an effective treatment regimen cannot otherwise be composed for reasons of resistance or tolerability (10). The recommended dosage of delamanid is 100 mg twice daily (BID) for 6 months.

Pharmacokinetic considerations play a particularly prominent role in the development and analysis of TB pharmacotherapies, given the complexities of current combination regimens, the extended nature of the treatment periods, and the underlying refractoriness of the infection itself. In the case of delamanid, pharmacokinetic studies have found that steady-state exposure to drug was less than proportional with increasing dose, i.e., a 2-fold increase from 100 mg BID to 200 mg BID resulted in a 1.5-fold increase in mean area under the daily concentration-time curve (AUC_0–24,ss_) (7,925 versus 11,837 h · ng/ml, respectively) (5). The elimination half-life was about 30 to 38 h (5), with albumin appearing to be predominantly responsible for metabolizing delamanid to its primary metabolite (11). No clinically relevant drug-drug interactions between delamanid and antiretroviral drugs or anti-TB drugs were observed in healthy subjects (12), but absorption of delamanid increased significantly when it was taken with food (13).

In the present study, we developed a population pharmacokinetics model aiming to (i) characterize delamanid pharmacokinetics following oral administration of various dosing regimens in patients with pulmonary MDR-TB (including estimation of variability in delamanid exposure); (ii) identify and quantify intrinsic and extrinsic factors (e.g., demographics, lab values, TB and HIV status, delamanid dose, administration conditions, and drug interactions) that may alter delamanid exposure; and (iii) simulate pharmacokinetics (PK) data following alternative dosing regimens, in particular 200-mg once-daily (QD) dosing.

## RESULTS

### Data.

Patients were pooled from four delamanid clinical studies: three phase II trials (trials 204, 208, and 210) and one phase III trial (trial 213) (see Table 1 for key features of included trials). Overall, the data set included 20,483 plasma samples from 744 patients (321, 10, and 339 patients from trials 204, 210, and 213, respectively; in addition, the analysis included 74 patients who had switched from placebo to delamanid in trial 208). All except 10 patients received delamanid in combination with an optimized background regimen (OBR). Delamanid oral dosing regimens assessed in the studies included 100 mg BID for 8 weeks and 200 mg BID for 8 weeks (trial 204), 250 mg BID for 28 weeks and 300 mg BID for 28 weeks (trial 210), and 100 mg BID for 8 weeks followed by 200 mg QD in the morning for 18 weeks (trial 213). In addition, patients who successfully completed treatment with 100 mg delamanid BID, 200 mg delamanid BID, or matching placebo in trial 204 could enter trial 208 and receive delamanid for an additional 26 weeks at a starting dose of 100 mg BID, with an option to titrate to 200 mg BID after 2 weeks. In all studies, delamanid was required to be taken with food and under direct observation for the morning dose.

**TABLE 1:** Summary of delamanid clinical trials examined in this study^*a*^

Study	Description	PK sampling schedule
Trial 204 (5) (242-07-204; NCT00685360), *n* = 481	Phase II, multicenter, randomized, double-blind, stratified (by extent of pulmonary MDR-TB), placebo-controlled trial. Patients were randomized 1:1:1 to receive delamanid 100 mg BID, delamanid 200 mg BID, or placebo BID plus OBR for 8 wk.	Blood samples were collected on days 1, 14, 28, and 56 predose and at 2, 3, 4, 10, 12, 13, 14, and 24 h postdose, and on days 63, 70, 77, 84.
Trial 208 (6) (242-07-208; NCT02573350), *n* = 421	Phase II, multicenter, uncontrolled, open-label trial. Patients who successfully completed trial 204 (above) were treated with delamanid plus OBR for up to an additional 26 wk. All patients received an initial delamanid dose of 100 mg BID, with an option to titrate to 200 mg BID afterward.	Blood samples were obtained at baseline (before the first dose) and wk 2, 6, 10, 14, 18, 22, and 26.
Trial 210 (242-08-210; NCT01131351), *n* = 10	Phase II, multicenter, noncontrolled, nonrandomized, open-label trial. Two cohorts of 5 patients each received 250 mg BID and 300 mg BID delamanid, respectively, for 28 wk.	Blood samples were collected on days 1, 14, 28, 56, 112, and 196 predose and at 3, 6, 9, 12, and 24 h postdose.
Trial 213 (9) (242-09-213; NCT01424670), *n* = 511	Phase III, multicenter, randomized, double-blind, stratified, placebo-controlled trial. Delamanid was administered as 100 mg BID for 8 wk followed by 200 mg QD in the morning for 18 wk. HIV-positive patients on anti-retroviral therapy (*n* = 48) were enrolled in an HIV subtrial.	Blood samples were collected on day 1 and wk 2, 4, 6, 8, 10, 12, 14, 16, 18, 20, 22, 24, and 26 predose and 2–8 h postdose.

a All patients in the trials had pulmonary MDR-TB. BID, twice daily; MDR-TB, multidrug-resistant tuberculosis; OBR, optimized background regimen; QD, once daily.

Of the 744 patients included, 70.4% had MDR-TB, 16.8% had pre-XDR-TB (pre-extensively drug-resistant TB, i.e., resistance to isoniazid, rifampin, and either second-line injectables or fluoroquinolones), and 12.8% had XDR-TB (resistance to isoniazid, rifampin, second-line injectables, and fluoroquinolones; pre-XDR-TB and XDR-TB status was unknown for 109 MDR-TB patients) (Table 2). Asian (40.5%) and white (23.8%) patients made up the majority of the population. Among all patients, 4.2% were coinfected with HIV. Also, 27.25% of patients had baseline serum albumin values below 3.4 g/dl, and 8.7% had baseline serum albumin levels at or below 2.8 g/dl. Mild and moderate renal impairment were observed in 15.3% and 1.6% of patients, respectively. Among all patients, 2.6% had baseline alanine aminotransferase (ALT) values above 56 U/liter, 13.7% had baseline aspartate aminotransferase (AST) values above 40 U/liter, 1.6% had baseline AST values above 80 U/liter, and 2.2% had baseline total bilirubin levels of >1 mg/dl (Child-Pugh scores were not assessed).

**TABLE 2:** Demographics and baseline characteristics of the pooled study population^*a*^

Characteristic	Value
Total patients, *n*	744
Total plasma samples, *n*	20,483
Male, *n* (%)	517 (69.5)
Race, *n* (%)	
Asian	301 (40.5)
Southeast Asia (Philippines)	200 (26.9)
Northeast Asia (China, Japan, or Korea)	99 (13.3)
Non-Asian (Peru)	2 (0.3)
White	177 (23.8)
Black	51 (6.9)
Other	215 (28.9)
Median age, yr (range)	33 (18–64)
Median body wt, kg (range)	55 (27–99.6)
Tuberculosis status, *n* (%)	
MDR-TB	524 (70.4)^*b*^
Pre-XDR-TB	125 (16.8)
XDR-TB	95 (12.8)^*b*^
HIV-positive, *n* (%)	31 (4.2)
Efavirenz-treated	22 (3.0)
Lamivudine-treated	23 (3.1)
Tenofovir-treated	16 (2.2)
Serum albumin level, *n* (%)	
<3.4 g/dl	202 (27.2)
<2.8 g/dl	65 (8.7)
Glomerular filtration rate,^*c*^ *n* (%)	
CKD stage II (≥60 and <90 ml/min/1.73 m^2^)	114 (15.3)
CKD stage III (≥30 and <60 ml/min/1.73 m^2^)	12 (1.6)
Hepatic markers, *n* (%)	
ALT > 56 U/liter	19 (2.6)
AST > 40 U/liter	102 (13.7)
AST > 80 U/liter	12 (1.6)
Total bilirubin > 1 mg/dl	16 (2.2)

a CKD, chronic kidney disease; MDR-TB, multidrug-resistant tuberculosis; ULN, upper limit of normal; XDR-TB, extensively drug-resistant tuberculosis.

b XDR-TB status was unknown for 109 of 524 patients in the MDR-TB group.

c According to the Modification of Diet in Renal Disease (MDRD) Study equation.

The drugs that constituted the concomitant OBR and were administered to more than 10% of patients in one of the trials (except trial 210; as there were only 10 patients in trial 210, this trial’s data did not influence the inclusion decision) were included in modeling (Table 3). Amikacin and gatifloxacin were also included, although they did not make the cutoff of 10% (they were administered in 7% and 8% of patients in trials 204 and 208, respectively).

**TABLE 3:** Numbers of patients on concomitant tuberculosis medications for 80% of the delamanid treatment duration^*a*^

Drug	No. (%) of patients in trial:			
	204	208^*b*^	210	213
AMIKA	16 (5)	14 (7)	0 (0)	46 (14)
AMINO	109 (34)	92 (43)	3 (30)	61 (18)
AMOXI	25 (8)	34 (16)	0 (0)	3 (1)
CAPRE	59 (18)	35 (16)	1 (10)	27 (8)
CYCLO	255 (79)	134 (63)	10 (100)	267 (79)
ETHAM	172 (54)	84 (39)	6 (60)	136 (40)
ETHIO	94 (29)	29 (14)	0 (0)	150 (44)
GATIF	15 (5)	16 (8)	0 (0)	0 (0)
ISONI	27 (8)	33 (15)	0 (0)	11 (3)
KANAM	172 (54)	14 (7)	2 (20)	159 (47)
OFLOX	290 (90)	171 (80)	2 (20)	256 (76)
PROTI	191 (60)	127 (60)	2 (20)	70 (21)
PYRAZ	169 (53)	91 (43)	1 (10)	213 (63)
STREP	37 (12)	3 (1)	0 (0)	0 (0)
MOXIF	0 (0)	0 (0)	0 (0)	54 (16)

a AMIKA, amikacin; AMINO, *p*-aminosalicylic acid; AMOXI, amoxicillin; CAPRE, capreomycin; CYCLO, cycloserine; ETHAM, ethambutol; ETHIO, ethionamide; GATIF, gatifloxacin; ISONI, isoniazid; KANAM, kanamycin; MOXIF, moxifloxacin; OFLOX, ofloxacin or levofloxacin; PROTI, protionamide; PYRAZ, pyrazinamide; STREP, streptomycin.

b A sample size of *n* = 208 was used for calculation of percentages for trial 208, which included not only the 74 patients who switched from placebo to delamanid but also those who were originally on delamanid in trial 204.

### Pharmacokinetic model of delamanid.

The pharmacokinetics of delamanid in patients with MDR-TB following various oral regimens was best characterized by an extravascular two-compartment model with first-order elimination and absorption, an absorption lag time, and decreased relative bioavailability with increasing dose. The model was parameterized in terms of the following variables: relative bioavailability (*F*_1_), where a 100-mg dose administered in the morning in non-Asian subjects in an inpatient setting was used as the reference; apparent clearance (CL/*F*); apparent volumes of distribution of the central and peripheral compartments (*V*_2_/*F* and *V*_3_/*F*); apparent intercompartmental clearance (*Q*/*F*); first-order absorption rate constant (separate for morning and afternoon doses; *K_a_*_,AM_ and *K_a_*_,PM_); and absorption lag time (also separate for morning and afternoon doses, LAG_AM_ and LAG_PM_). The parameters CL/*F*, *V*_3_/*F*, *K_a_*_,AM_, *K_a_*_,PM_, *Q*/*F*, and *F*_1_ had an associated log-normally distributed interindividual variability. The residual variability was represented by a combined additive and proportional error model.

**FIG 1:**
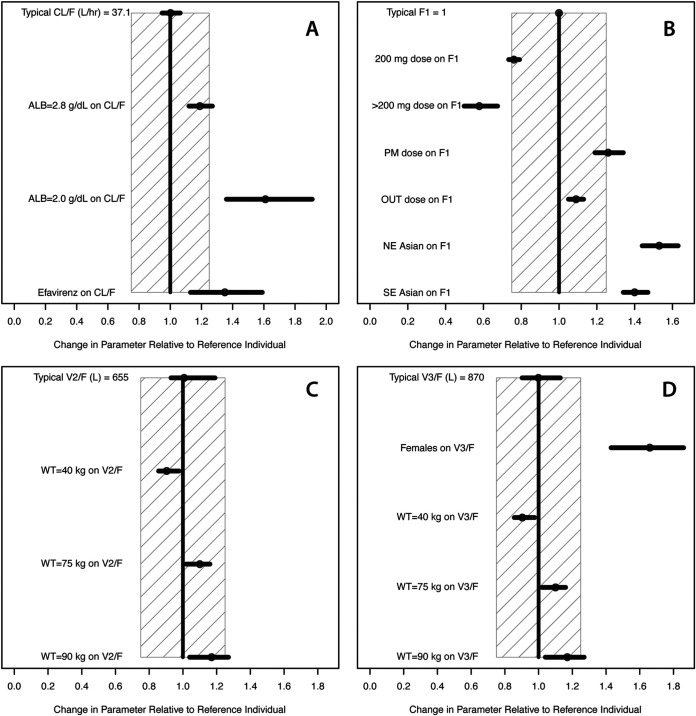
Dependence of delamanid pharmacokinetic parameters on covariates in the final model. For the typical parameter value, the circle and the horizontal line indicate the ratio of the bootstrap median and 95% confidence interval to the typical value of the model parameter. For the covariate effects, circles and horizontal lines indicate bootstrap median and 95% confidence intervals, respectively. Boundaries of the shaded areas indicate a 25% change in the parameter value. For panel A, the typical patient was a 55-kg non-Asian male with serum albumin at baseline of ≥3.4 g/dl who did not receive efavirenz; for panel B, the typical patient was a non-Asian who received a 100-mg morning dose as an inpatient; for panels C and D, the typical patient was a 55-kg male. ALB, serum albumin at baseline; CL/*F*, apparent clearance; *F*_1_, relative bioavailability; OUT, outpatient setting; *V*_2_/*F*, apparent central volume; *V*_3_/*F*, apparent peripheral volume; WT, weight.

The parameter estimates of the final model are provided in Table 4. The influence of covariate effects on delamanid PK parameters is illustrated in Fig. 1. After the prespecified covariate modeling process, the following covariate effects were identified and retained in the final model. The absorption rate constant was higher following morning doses than evening doses (0.397 [95% confidence interval {CI}, 0.348 to 0.447] versus 0.248 [95% CI, 0.215 to 0.281] h^−1^, respectively), while the absorption lag time was shorter following morning doses than evening doses (0.825 [95% CI, 0.814 to 0.837] versus 1.38 [1.3 to 1.45] h, respectively). Relative bioavailability (*F*_1_) was 26% (95% CI, 22 to 29%) higher after evening doses than morning doses and 9% (95% CI, 8 to 10%) higher in outpatient settings than inpatient settings. Relative bioavailability of the 200-mg dose was estimated to be 24.0% (95% CI, 22.8 to 25.1%) lower than that of the 100-mg dose. For doses higher than 200 mg (250 mg and 300 mg), relative bioavailability was estimated to be 42.0% (95% CI, 32.4 to 51.2%) lower than that of the 100-mg dose. Relative bioavailability was 53% (95% CI, 43 to 63%) higher in northeast Asian patients and 40% (95% CI, 32 to 48%) higher in southeast Asian patients than in non-Asian patients.

**TABLE 4:** Parameter estimates for the final model^*a*^

PK parameter	Parameter	Estimate (95% CI)	RSE (%)	Bootstrap (median [95% CI])	Variability (CV [%])	Shrinkage (%)
CL/*F* (liters/h)	θ_1_	37.1 (35.8–38.4)	1.78	37.2 (35.2–39.5)		
*V*_2_/*F* (liters)	θ_2_	655 (604–707)	4.02	660 (609–780)		
Q/*F* (liters/h)	θ_3_	104 (90.1–117)	6.66	105 (92.9–137)		
*V*_3_/*F* (liters)	θ_4_	870 (792–948)	4.58	869 (785–982)		
*K_a_*_,AM_ (1/h)	θ_5_	0.397 (0.348–0.447)	6.38	0.409 (0.364–0.89)		
LAG_AM_ (h)	θ_6_	0.825 (0.814–0.837)	0.683	0.826 (0.791–1.58)		
*F*_1, 200 mg_	θ_7_	0.760 (0.749–0.772)	0.771	0.761 (0.734–0.791)		
*F*_1, >200mg_	θ_8_	0.580 (0.483–0.676)	8.51	0.578 (0.498–0.675)		
*K_a_*_,PM_ (1/h)	θ_9_	0.248 (0.215–0.281)	6.79	0.251 (0.232–0.293)		
LAG_PM_ (h)	θ_10_	1.38 (1.3–1.45)	2.84	1.38 (1.31–1.44)		
*F*_1,PM_	θ_11_	1.26 (1.22–1.29)	1.36	1.26 (1.19–1.34)		
*F*_1,out_	θ_12_	1.09 (1.08–1.10)	0.658	1.09 (1.05–1.13)		
*V*_2_, *V*_3,WT_	θ_13_	0.316 (0.162–0.47)	24.8	0.318 (0.0742–0.489)		
*V*_3,SEX_	θ_14_	1.65 (1.45–1.85)	6.29	1.66 (1.43–1.86)		
*F*_1,NEAsian_	θ_15_	1.53 (1.43–1.63)	3.29	1.53 (1.44–1.63)		
*F*_1,SEAsian_	θ_16_	1.40 (1.32–1.48)	2.92	1.40 (1.34–1.47)		
CL_ALB<3.4_^*b*^	θ_17_	−0.892 (−1.03 to −0.756)	7.79	−0.899 (−1.22 to −0.583)		
CL_efavirenz_	θ_18_	1.35 (1.23–1.47)	4.57	1.35 (1.13–1.59)		
${\text{ω}}_{\text{CL}}^{2}$	Ω(1,1)	0.056 (0.0464–0.0656)	8.78	0.0563 (0.0422–0.0724)	23.7	19.6
${\text{ω}}_{V3}^{2}$	Ω(2,2)	0.152 (0.129–0.174)	7.58	0.150 (0.063–0.401)	39.0	44.7
${\text{ω}}_{\mathrm{Ka}\text{AM}}^{2}$	Ω(3,3)	0.517 (0.424–0.61)	9.16	0.527 (0.422–0.71)	71.9	24.8
${\text{ω}}_{F1}^{2}$	Ω(4,4)	0.0344 (0.0244–0.0444)	14.8	0.0334 (0.0226–0.045)	18.5	33.9
${\text{ω}}_{Q}^{2}$	Ω(5,5)	0.456 (0.29–0.622)	18.6	0.471 (0.286–0.806)	67.5	49.2
${\text{ω}}_{KaP\text{M}}^{2}$	Ω(6,6)	0.343 (0.251–0.435)	13.7	0.35 (0.257–0.469)	58.6	42.5
${\text{σ}}_{\text{pr}}^{2}$	Σ(1,1)	0.0715 (0.0706–0.0724)	0.638	0.0709 (0.0652–0.0762)	26.7	4.3
${\text{σ}}_{\text{pr,7208}}^{2}$	Σ(2,2)	0.174 (0.163–0.186)	3.45	0.176 (0.15–0.204)	41.8	2.3
${\text{σ}}_{\text{add,9213}}^{2}$ (ng/ml)²	Σ(3,3)	1,950 (1880–2020)	1.92	1,950 (1650–2290)	44.2^*c*^	3.3
${\text{σ}}_{\text{add}}^{2}$ (ng/ml)²	Σ(4,4)	2.39 (2.05–2.73)	7.26	2.38 (0.399–6.86)	1.55^*c*^	4.8

a PE, parameter estimate; SE, standard error; RSE, relative standard error (100 × SE/PE); 95% CI, 95% confidence interval; SD, standard deviation; CV, coefficient of variation (100 × SD%).

b CL/*F* ∼ IALB^θ17^, where IALB is 1 if ALB is ≥3.4 g/dl and IALB is ALB/3.4 if ALB is ≤3.4 g/dl.

c Standard deviation.

Apparent clearance (CL/*F*) was independent of body weight, while apparent central volume of distribution (*V*_2_/*F*) and apparent peripheral volume of distribution (*V*_3_/*F*) were slightly higher at higher weight (power coefficient, 0.316; 95% CI, 0.162 to 0.47). The apparent peripheral volume of distribution in female patients was 65% (95% CI, 45 to 85%) higher than in male patients. Apparent clearance was higher in patients with baseline serum albumin concentration below 3.4 g/dl, proportionally to the power function with the power coefficient of −0.892 (95% CI, −1.03 to −0.756). At a baseline albumin level of 2.8 g/dl, apparent clearance was higher by about 22%, implying a decrease of about 18% in delamanid exposure. Further increase in delamanid clearance occurred at baseline albumin levels below 2.8 g/dl, although it should be noted that only 65 subjects had baseline albumin levels this low in the trials. Delamanid pharmacokinetics was not influenced by the patient’s HIV status. However, apparent clearance was 35% (95% CI, 23 to 47%) higher in patients with HIV who were coadministered efavirenz but not other antiretroviral therapies.

Covariates that were considered but not retained in the final model included age, XDR status, total serum protein level, estimated creatinine clearance or other measures of renal function (estimated normalized creatinine clearance (ml/min/1.73 m^2^) and Modification of Diet in Renal Disease Study [MDRD] equation), measures of hepatic function (alanine aminotransferase, aspartate aminotransferase, total bilirubin, and alkaline phosphatase), individual components of OBR therapy, concomitant CYP 3A4 inducers and inhibitors (grouped as classes), and concomitant gastrointestinal agents (grouped as a class).

The interindividual variability of delamanid PK parameters was low for CL/*F* and *F*_1_ (coefficient of variation [CV] = 23.7% and 18.5%, respectively), moderate for *V*_3_/*F* (CV = 39%), and high for *Q*/*F* and absorption rate constants (*K_a_*_,AM_ and *K_a_*_,PM_; CV = 71.9% and 58.6%, respectively). The proportional part of the residual variability was higher in trial 208 (CV = 41.8%) than in the other trials (CV = 26.7%), while the additive part of the residual variability was higher in trial 213 (standard deviation [SD] = 44.2 ng/ml) than in the other trials (SD = 1.55 ng/ml).

### Model evaluation.

**FIG 2:**
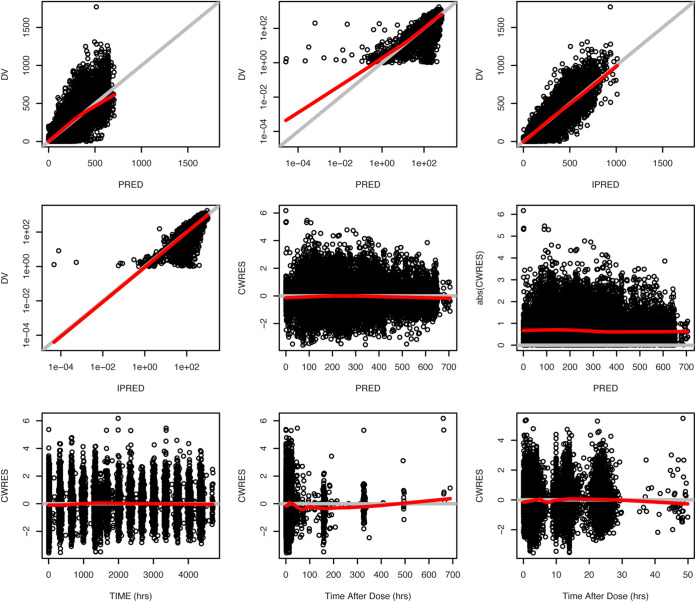
Goodness-of-fit plots for the final model (model 013). Shown are data from all patients. Bold red lines are the lowess (local regression smoother) trend lines; gray lines (*y* = *x* or *y* = 0) are included for reference. DV, observed concentrations; PRED, population predictions of the model; IPRED, individual predictions of the model; CWRES, conditional weighted residuals; TIME, time after the first dose; TAD, time after the most recent dose. DV versus PRED and DV versus IPRED plots are presented both in linear and log scales.

**FIG 3:**
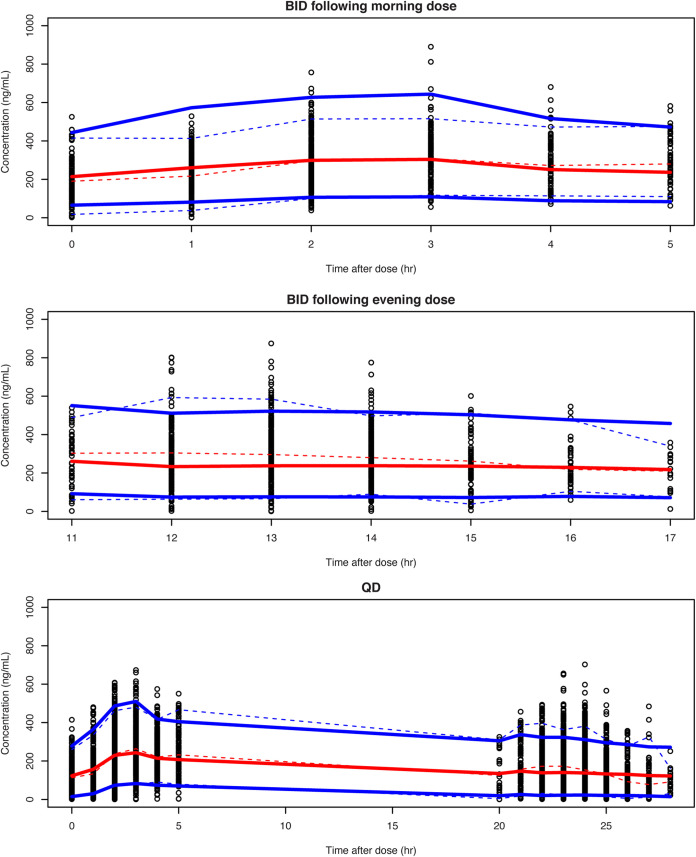
Visual predictive check of the final model (trial 242-09-213). The dashed and solid lines show the median (red) and the 5th and 95th percentiles (blue) of the observed and simulated values, respectively. The simulated values were computed from 1,000 trials simulated using dosing, sampling, and the covariate values of the analysis data set. BID, twice-daily dosing; QD, once-daily dosing.

All structural parameters, covariate effects, and variance parameters were well estimated in the final model. Goodness-of-fit plots (Fig. 2) and visual predictive check plots (Fig. 3) indicated reasonable model fit and acceptable predictability of the final model. There were no dependencies of the random effects on any of the covariates after retention of the above-discussed significant covariates in the final model. Bootstrap parameter distributions (from 1,000 bootstrap runs) were consistent with the final model parameter estimates (Table 4).

As described in Materials and Methods, model building in this study occurred in two stages: an initial model was developed in stage 1 using data from trials 204, 208, and 210, and the final model in stage 2 included additional data from trial 213 (Table 1). All pharmacokinetic parameter estimates of the final model were consistent with the initial model, providing additional evidence of the robustness, consistency, and validity of the results.

### Model application.

**FIG 4:**
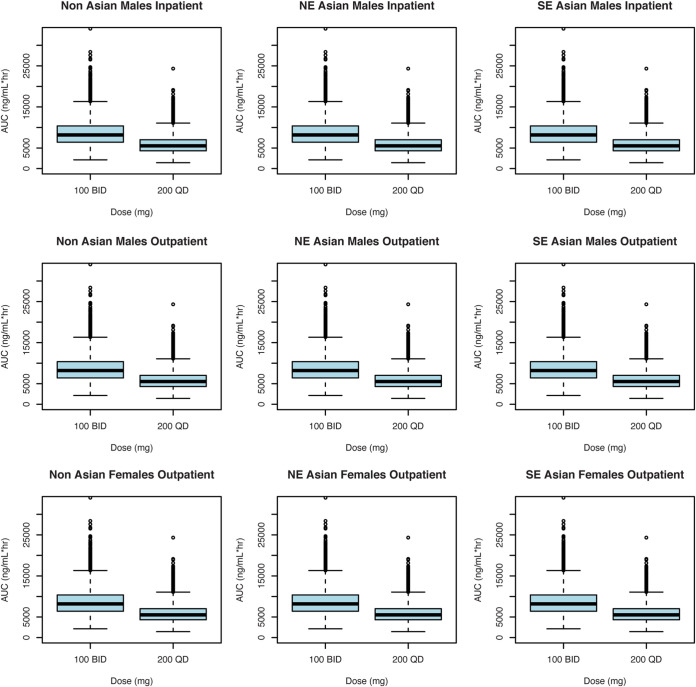
Predicted distributions of delamanid steady-state AUC_0–24_ for 100-mg BID versus 200-mg QD dosing. The values were simulated from the final model for 2,000 patients weighing 55 kg, without hypoalbuminemia, not coadministered efavirenz, and with other covariate values shown on the plots. Concentrations were simulated for each hour for 24 h. Residual variability was not included. Median values are designated by black lines in the centers of the boxes. Boxes indicate the interquartile range (IQR). Whiskers represent 1.5× IQR. Outliers are marked outside the whiskers by circles.

The final model was used to predict concentration-time profiles and steady-state exposures of delamanid after 100-mg BID dosing for 20 days, followed by 200-mg QD morning dosing, in identified subpopulations (non-Asian, northeast Asian, or southeast Asian; male or female; inpatient or outpatient) (Table 5 and Fig. 4). Total daily exposure at steady state (AUC_0–24,ss_) was approximately 1.5-fold higher in patients who received the 100-mg BID dose than in those who received the 200-mg QD dose (Table 5 and Fig. 4). Delamanid accumulation for the 100-mg BID dose, defined as the ratio of AUC_0–24_ at steady state to that on day 1, was in the range of 3.1 to 3.4. Exposure in Asian patients was higher than in non-Asian patients, while outpatient exposure was slightly higher than inpatient exposure.

**TABLE 5:** Predicted delamanid *C*_max_ and AUC_0–24_ at steady state following 100-mg BID or 200-mg QD morning dosing regimens^*a*^

Dosing regimen	Geographic region	Gender	*C*_max_ (ng/ml) (mean CV [%])	AUC_0–24_ (ng/ml · h) (mean CV [%])
100 mg BID	Non-Asian	Male	333 (28)	6,863 (31)
		Female	334 (29)	6,879 (31)
	Northeast Asian	Male	516 (30)	10,673 (32)
		Female	511 (28)	10,488 (30)
	Southeast Asian	Male	474 (29)	9,812 (31)
		Female	464 (29)	9,551 (30)

200 mg QD morning	Non-Asian	Male	258 (28)	4,625 (31)
		Female	263 (29)	4,665 (31)
	Northeast Asian	Male	400 (30)	7,194 (32)
		Female	400 (28)	7,116 (31)
	Southeast Asian	Male	366 (29)	6,611 (31)
		Female	364 (29)	6,479 (31)

a Predictions are based on simulations for 2,000 MDR-TB patients weighing 55 kg who were not hypoalbuminemic and were not coadministered efavirenz in outpatient settings. Residual variability was not included in the simulations. AUC_0–24_, daily area under the concentration-time curve; *C*_max_, maximum drug concentration; CV, coefficient of variation.

## DISCUSSION

The present study assessed the population pharmacokinetics of delamanid in patients with MDR-TB. The final model was constructed based on data from four clinical trials (*n* = 744). The data were primarily from trials 204, 208, and 213 (100 mg BID, 200 mg BID, and 200 mg QD). In addition, data were included from 10 patients enrolled in trial 210 (250 mg BID and 300 mg BID) (Table 1). Nearly all patients in the combined study population (*n* = 734) received delamanid in combination with various OBR treatments, and all patients were required to receive delamanid with food. Depending on the individual study, patients were hospitalized either all of the time, part of the time, or none of the time during delamanid treatment, which also allowed an exploration of differences in delamanid pharmacokinetics in outpatient versus inpatient settings.

Concentration-time profiles of delamanid were adequately described for all observed dosing conditions and subpopulations by a two-compartment model with first-order elimination and absorption, an absorption lag time, and decreased relative bioavailability with increasing dose (Table 4). Graphical and simulation-based (visual predictive check) evaluations of the final model, as well as bootstrap analyses, indicated a reasonable model fit and adequate predictive power, and final parameters in the model were consistent with prior published pharmacokinetic analyses of delamanid (5, 11, 12, 14). The following factors had no significant effects on delamanid exposure in patients with MDR-TB: age (range, 18 to 64 years); gender; weight (range, 27.0 to 99.6 kg); XDR-TB status; anti-TB OBR medications (amikacin, *p*-aminosalicylic acid, amoxicillin with clavulanic acid, capreomycin, cycloserine, ethambutol, ethionamide, gatifloxacin, isoniazid, kanamycin, levofloxacin or ofloxacin, moxifloxacin, protionamide, pyrazinamide, or streptomycin); lamivudine, tenofovir, or pyridoxine; CYP 3A4 inhibitors (isoniazid, cimetidine, rabeprazole, and ranitidine) and inducers (rifabutin, rifampin, rifapentine, pantoprazole, and omeprazole); gastrointestinal agents for acid-related disorders; HIV positivity; mild or moderate renal impairment; and hepatic function (as measured by ALT, AST, or total bilirubin levels).

Final results from the model showed that exposures were higher in Asian patients than non-Asian ones, and delamanid oral clearance increased in patients with albumin levels of <3.4 g/dl, thus implying a decrease in exposure. Coadministration of efavirenz to patients with HIV increased delamanid oral clearance by 35%, indicating an approximately 25% decrease in AUC_0–24_, which was not seen in a controlled trial with healthy subjects (12). However, none of these effects on delamanid PK were determined to be clinically relevant based on totality of data in the delamanid clinical program, and none of the changes in the factors examined in the current population PK analysis would therefore warrant a need for dose adjustment for delamanid. The higher exposure in Asian subjects compared to non-Asian subjects was also seen in phase I trials in healthy subjects. It is possible that differences in dietary habits between Asian and non-Asian regions, e.g., the fat content of food consumed around dosing, may explain the differences, since absorption of and exposure to delamanid are significantly affected by food, in particular fat content (15). Regarding the finding of increased clearance in patients with hypoalbuminemia, there is evidence that hypoalbuminemia leads to decreased protein binding and, thus, to increased total clearance (16). It is also possible that malabsorption in the hypoalbuminemic patients, who were likely malnourished, was a factor leading to decreased bioavailability of delamanid and higher apparent delamanid clearance (17).

Currently, delamanid is recommended at a dose of 100 mg twice a day (10), a safe and effective dosing regimen in adult patients with MDR-TB (5, 6, 9, 18, 19). However, given the complexity of MDR-TB therapy, which can involve multiple antibiotic agents administered over extended times, it would be of significant clinical interest to identify a once-daily form of delamanid that could be integrated more accessibly into a variety of complex anti-TB regimens. Trial 213 (Table 1) assessed the potential of a 200-mg dose of delamanid given only once per day (following 8 weeks of prior therapy at 100 mg BID) and found it to be safe (9). Complete characterization of the 200-mg QD dose in relation to the currently recommended 100-mg BID dose, however, requires a detailed understanding of the pharmacokinetic/pharmacodynamic relationship of delamanid, which is beyond the pharmacokinetic scope of this study but was explored in a separate analysis (20).

This report has several potential limitations. First, patients in the included trials were 18 to 64 years old, and thus, the effects of older ages (>65 years) on delamanid PK parameters were not explored. Second, as described above, absorption rates were higher and absorption lag times were shorter following morning doses of delamanid than evening doses, but it remains unclear whether this observation reflected a genuine physiological mechanism (e.g., dawn effect) or merely a difference in the amount and/or composition of food consumed in the morning and in the evening, considering the significant food effect observed with delamanid. Third, the exact reason(s) for the observed differences between Asian and non-Asian populations, while likely related to food intake and/or the fat content of food, remains unclear. Fourth, the numbers of patients contributing data at the highest dosages of delamanid (250 mg BID and 300 mg BID) were small (*n* = 5 in each dosing cohort). It is noteworthy, however, that patient numbers were much higher for the lower and more clinically relevant dosages; moreover, several of the trials assessing lower dosages included rich pharmacokinetic sampling, allowing a more granular assessment of PK parameters than would have been possible with only sparse PK sampling. Lastly, the generalizability to other populations with a high burden of MDR-TB, such as sub-Saharan African patients, is unknown.

In summary, this report showed that concentration-time profiles of delamanid were best described by a two-compartment model with first-order elimination and absorption, an absorption lag time, and decreased relative bioavailability with increasing dose. Pharmacokinetic parameters for delamanid appeared to be robust across multiple tested factors and, thus, no conditions have been identified to date that would require dose adjustment. Model evaluation suggested reasonable model fit and adequate predictive power, providing confidence that the model should prove reliable for deriving PK metrics in subsequent PK/PD analyses of delamanid and its role in the treatment of MDR-TB.

## MATERIALS AND METHODS

### Data.

Data for the population PK analyses were pooled from four delamanid clinical trials conducted in patients with pulmonary MDR-TB, i.e., trial 204 (NCT00685360), trial 208 (NCT02573350), trial 210 (NCT01131351), and trial 213 (NCT01424670). All four trials were supported by Otsuka America Pharmaceutical, Inc. (Rockville, MD, USA), and were in compliance with International Conference on Harmonization and good clinical practice guidelines for conducting, recording, and reporting clinical trials, as well as for archiving essential documents. No trial procedures were performed on trial candidates until written consent had been obtained. The informed-consent form, protocol, and amendments for the study were submitted to and approved by the institutional review board or independent ethics committee for each respective trial site or country.

Essential design features of the four trials are described in Table 1.

### Model development.

Model building was performed in two stages: an initial model was developed in stage 1 using data from trials 204, 208, and 210. To be included in the stage 1 analysis, concomitant medications were to be taken by at least 10% of patients for at least 80% of the trial duration. In stage 2, additional data from trial 213 was added to the analysis.

Population PK analysis was conducted during stages 1 and 2 via nonlinear mixed-effects modeling with NONMEM software, version 7.2 (ICON Development Solutions, Ellicott City, MD, USA) (21). Graphical and all other statistical analyses, including evaluation of NONMEM outputs, were performed using R (22). The first-order conditional estimation with interaction (FOCEI) method in NONMEM was employed for all model runs.

Pretreatment plasma samples and posttreatment concentrations below the lower limit of quantification were excluded from the analysis, as were concentration values grossly inconsistent with concentration profiles (outliers).

### (i) Stage 1.

One-, two- and three-compartment linear models with first-order absorption and absorption lag time were initially implemented and compared. Individual concentration profiles, model fit, and individual model parameters strongly depended on administered dose and dosing conditions (e.g., morning or evening, inpatient or outpatient), and therefore, the influence of dosing conditions on delamanid PK was explored at the stage of base modeling.

All interindividual variability terms were described by a log-normal parameter distribution:

Pi=P^exp(ηPi)

where *P_i_* is the estimated parameter value for individual *i*, P^ is the typical population value (geometric mean) of the parameter, and ${\text{η}}^{P_{i}}$ represents individual-specific interindividual random effects for individual *i* and parameter *P*. The random effects were assumed to be normally distributed (η ∼ *N*[0, ω^2^]), with covariances defined by the interindividual covariance matrix Ω. When necessary and justified by the data, the block-diagonal or full covariance matrix Ω for the interindividual random effects was used.

For concentration values in this analysis, the residual error model was described by a combined proportional and additive error:

Cij=C^ij(1+ωprεij1)+ωaddεij2

where *C_ij_* is the *j*th measured observation in individual *i*, C^ij is the *j*th model-predicted value in individual *i*, ${\text{ω}}_{\text{pr}}^{2}$ and ${\text{ω}}_{\text{add}}^{2}$ are variances of the proportional and additive components of the residual random error, respectively; and ${\text{ε}}_{\mathrm{ij}}^{1}$, ${\text{ε}}_{\mathrm{ij}}^{2}$, and ε*_ij_* are the residual errors for individual *i* and measurement *j* assumed to be independently and identically distributed according to the standard normal distribution (ε ∼ *NID*[0,1]).

Following the establishment of the base model, a covariate model was developed.

The effects of continuous covariates were modeled multiplicatively using a normalized power model:

$${\text{TVP}}_{i}={\text{θ}}_{1}\times {\left(\frac{{\text{COV}}_{i}}{{\text{COV}}_{\text{REF}}}\right)}^{{\text{θ}}_{2}}$$

where TVP*_i_* is the typical value of a PK parameter (P) for individual *i* with the value of the covariate COV*_i_* and θ_1_ is the typical value for an individual with the reference covariate value COV_REF_.

For categorical covariates, the fractional change in the typical parameter value was determined, and covariates were introduced multiplicatively in the model.

The covariate modeling utilized the full-model approach (23, 24). Potential covariate-parameter relationships were identified based on scientific interest, mechanistic plausibility, and exploratory graphics, as well as on the range, distributions, and correlations of covariate values in the data set. The considered covariates (model component[s]) were weight and/or other body size measures (all clearance and volume parameters); gender (clearance, volume, and bioavailability); age (clearance); race and/or geographical region and ethnicity (clearance, volume, and bioavailability); XDR status (clearance and volume); serum albumin level (clearance and volume); total serum protein level (clearance); estimated creatinine clearance or other measures of renal function, e.g., estimated normalized creatinine clearance and Modification of Diet in Renal Disease Study equation (clearance); measures of hepatic function, e.g., alanine aminotransferase, aspartate aminotransferase, total bilirubin, and alkaline phosphatase levels (clearance); individual components of OBR therapy (clearance and bioavailability); concomitant CYP 3A4 inducers and inhibitors, grouped as classes (clearance and bioavailability); concomitant gastrointestinal agents, grouped as a class (clearance and bioavailability); HIV infection (clearance and bioavailability); and individual concomitant HIV medications (clearance and bioavailability).

All potential covariate-parameter relationships were entered in the model simultaneously, and parameters were estimated. The full model did not simultaneously include effects of strongly correlated or colinear predictors; when necessary, several competing full models were considered.

Inferences about covariate effects and their clinical relevance were based on the parameter estimates and measures of estimation precision (95% confidence intervals based on asymptotic standard errors). This approach allowed the direct assessment of clinical relevance of covariate effects and also provided some explanation for the apparent absence of a covariate effect (true lack of the effect versus lack of information about that effect). Specifically, interpretation and further refinement of the covariate model was based on point estimates, confidence intervals, and diagnostic plots of the covariate effects. Small (clinically insignificant) and precisely estimated effects were excluded to arrive at a parsimonious model.

Due to the large number of covariates considered, covariate modeling was performed in three steps. First, intrinsic factors such as body size measures, demographics, patient XDR-TB resistance status, and baseline clinical laboratory values were entered in the full model. If a covariate effect on a parameter was <10% and the 95% confidence interval of the effect was contained within 25% of the null value, the covariate effect was eliminated. Next, extrinsic factors (i.e., all concomitant medications) were added to the model, and the same criteria were used to eliminate the covariates. Finally, following elimination of all nuisance covariates, clinically insignificant covariates were eliminated. The covariate was assumed to be potentially clinically significant if its effect on the relevant parameter exceeded 15% or the 95% confidence interval was not contained within 20% of the null value.

### (ii) Stage 2.

The final model from stage 1 was used as the starting model for stage 2.

First, visual predictive checks were performed for the new data (data from trial 213) using the stage 1 model. The model was then refitted to the combined data set. Previously tested covariate effects were evaluated graphically. The effects of HIV-related covariates (baseline HIV status and individual antiretroviral therapies [efavirenz, lamivudine, and tenofovir]), as well as additional OBR medication (moxifloxacin was administered to >10% of patients for at least 80% of the delamanid treatment time) not tested in stage 1 were tested in the model using the forward addition procedure. Additional covariate effects were accepted if they resulted in a significant OFV (objective function value) drop at an α significance level of 0.01 (6.84 points).

### Model evaluation.

The final model was evaluated by assessing goodness-of-fit plots, the precision, distributions, and correlations of parameter estimates via bootstrapping, and visual predictive checks for all data, stratified by trials, dosing regimens, and covariates.

### Model application.

The final model was used to predict concentration-time profiles and steady-state exposures of delamanid after 100-mg BID dosing for 20 days, followed by 200-mg QD morning dosing, in identified subpopulations (non-Asian, northeast Asian, or southeast Asian; male or female; inpatient or outpatient). The simulations were conducted using the final model for 2,000 patients weighing 55 kg, without hypoalbuminemia, not coadministered efavirenz, and with other covariate values in the identified subpopulations. Population parameter estimates and interindividual variability were included in the simulations, and residual variability was not used. Concentrations were simulated for each hour for 24 h during the first and last days of BID and QD dosing.
